# Pathology of Fever, &c. Founded on the Blood's Vitality

**Published:** 1825-02

**Authors:** John Jones

**Affiliations:** Member of the Royal College of Surgeons, &c. London. Derby


*To the Editors of the London Medical and Physical Journal.*

Gentlemen,—I am induced to trouble you with the accompanying rationale of Fever, which appears to me not only satisfactorily to explain the phenomena of fever, but also those of many other diseases, and to be supported by physiological and pathological facts. Should this communication meet with your approbation, I will occasionally continue my observations on the subject, for insertion in your valuable Journal. Although the opinions I have advanced might be somewhat novel, if they tend in the slightest degree to forward the advancement of medical science, either by their own worth, or by eliciting useful matter from my professional brethren, my object will be obtained.

I am, Gentlemen, your most obedient servant,

JOHN JONES,

**Member of the Royal College of Surgeons, &c. London.**

*Derby; January* 1*st*, 1825.

### Art. II.—*Pathology of Fever, &c. founded on the Blood's Vitality.*

In investigating the phenomena of disease, it is necessary to have clear views of the laws which influence the animal economy, and by which the operations of life are accomplished: physiology, therefore, forms the basis of pathology, and by its assistance alone we are enabled to reason correctly on the nature of disease. The principle of vitality is enveloped in impenetrable mystery; its effects only admit of demonstration: all explanations, therefore, of what life *is*, must necessarily be conjectural, and every one is at liberty to form his own opinion respecting it. This mysterious power has occupied the attention of physiologists from the days of Hippocrates; numerous theories respecting it have been advanced, each of which has had its admirers for the day, and passed away in succession; and the science of physiology at present consists only in observing the various effects exhibited by the vital principle in organised bodies, and in ascertaining the laws by which these effects are governed.

Vitality has been considered a modification of electricity, and this opinion, which has of late been ably advocated by Dr. W. Philip, I am inclined to think will gain strength the more closely the subject is investigated. There is reason to suppose that galvanic influence is the result of a chemical union between electrical fluid and oxygen: might not a similar union, occurring in the pulmonic blood, give rise to those phenomena characterising life in the blood, which, through the medium of the nerves, is communicated to the system? And as, in the process of galvanism, heat is abundantly evolved, might not animal heat be the effect of that operation by which the vitality of the blood is communicated to every part of the animal machine? Thus life, considered as a galvanic process, commences in the blood, and is completed or rendered manifest by the nervous system. This hypothesis seems corroborated by Mr. Brodie's experiments to ascertain the influence of the brain on the action of the heart, &c., which prove that animal heat is intimately connected with the development of nervous energy. "When the influence of the brain is cut off, the secretion of urine appears to cease, and no heat is generated, notwithstanding the function of respiration and the circulation of the blood continues to be performed, and the usual changes in the appearance of the blood are produced in the lungs."

Although the doctrine of the blood's vitality has been almost generally received since it was so ably supported by the late Mr. John Hunter, and has for its founder the illustrious discoverer of the circulation, it is denied by Blumenbach, one of the most distinguished physiologists of the present day. "Humidity," he says, "is indeed necessary in the living solid for the exertion of vitality; but that vitality exists in the solid as solid, is proved by the well-known instances of animalcules and the seeds of plants, in which, although long dried, the vital principle is so entire, that they again live and germinate." The dryness of seeds, which are capable of germinating, must be considered relatively. In their dryest state, it cannot be denied that they contain moisture; and in this moisture, no doubt, consists their vitality. If Blumenbach could present us with a seed capable of germinating, from which all moisture had been abstracted by heat, there might be some weight in his argument. The learned translator of his "Institutions of Physiology," on the contrary, is a strenuous advocate for the blood's vitality. "That fluids are as susceptible of life as solids, I cannot doubt. There is no reason why they should not be so, although a person who has not thought on the subject may be as unable to conceive the circumstance, as a West Indian to conceive that water may by cold become solid. It is impossible to deny that at least the fluid which becomes the embryo possesses life, because it becomes an organised being: although some may, perhaps, contend that the male semen acts simply the part of a peculiar stimulus to the living fluid of the ovarian vesicle; others, that the life of the fluid of the ovarian vesicle is afforded by the male semen. However, Blumenbach, in his 'Commentatio de vi Vitali Sanguinis,' grants both male and female genital fluids to be alive, notwithstanding that he fancies his victory over the defenders of the blood's life so complete, that, like that of the unfortunate Carthagenian Dido, 'in ventos vita recessit.' It is as easy to conceive the blood to be alive as the ovarian fluid. The great assertor of the life of the blood is Mr. Hunter, and the mere adoption of the opinion by Mr. Hunter would entitle it to the utmost respect from me, who find the most ardent and independent love of truth, and the genuine stamp of professional genius, in every passage of his works."

The principle of life in the blood Hunter calls "materia vitæ diffusa," which, through the medium of the blood's fluidity, pervades every part of the animal machine, regulates its various functions, and repairs the waste occasioned by the operations of life. The phenomena of vital energy are only characteristic marks of the blood's vitality; for, without vascularity, life could not subsist. The nerves are the only visible organs, through the medium of which these phenomena are rendered manifest. Although contractility be seated in the mucous membrane, irritability in the muscular fibre, and sensibility in the nerves, as asserted by physiologists; and although peculiar modes of vital energy appear to regulate the functions of the various tissues of the body; yet these phenomena can only be considered, in reference to disease at least, as modifications of vital energy derived from the blood, and rendered manifest through the medium of the nerves.

In viewing the pathology of fever in connection with Hunter's "materia vitæ diffusa," this principle in the blood must be considered a *material agent*, which, like electrical fluid, is only known by its effects. Arterial blood is endowed with a larger proportion of it than venous, as, according to Hunter, the only sources from whence it can be derived are the chyle and respiration, particularly the latter. The brain is chiefly instrumental in the production of nervous energy, although the nerves themselves have an inherent power of acquiring it from the blood, independently of the brain, as is proved by this organ being sometimes wanting in a full-grown living fœtus. In postmortem examinations of persons who have died of typhus, the veins of the cerebrum in particular, and generally those also of the other principal internal organs, have been found gorged with black blood, doubtless indicating a previous state of venous congestion; by which term is to be understood a mechanical distention, or overloading, of the veins of any particular part with blood, which, from its slow progress in such distended vessels, appears to be thrown out of the circulation, and deprived of those changes produced by respiration, so essential to animal existence. The operations of the nervous and the vascular systems reciprocally depend on each other. The brain and nerves receive their peculiar vitality from the blood, and the vascular system is enabled to perform its important functions through the agency of nervous energy. The action of some morbific power on the brain, lungs, or cuticular organisation, by deranging their functions, causes a determination of blood to the internal parts. This morbific power might arise from depressing passions, contagion, putrid effluvia, exposure to cold, &c. and seems to weaken the energies of the system by a direct debilitating influence on the nerves; the action of the heart and arteries becomes enfeebled; the blood is tardily propelled through its vessels, and accumulates in the veins of the larger viscera, constituting congestions. This state of the internal organs is an additional cause of debility. The vital energies, being thus depressed by the united operations of direct sedative influence on the nerves and venous congestions, symptoms characterising the first stage of fever are induced, which are always indicative of diminished nervous energy. But, as the venous blood, when in a state of congestion, is in a measure thrown out of the circulation, it does not receive its wonted proportions of materia vitæ diffusa from the arterial blood; and an accumulation of this principle is the consequence, particularly if congestions are suddenly established. This acts as a powerful stimulus to the heart and arteries, which become unusually excited, and *re-action* is produced, marking the commencement of the second stage.

In all fevers, re-action alternates more or less with a state of depression, the degree of which is in proportion to the violence of the preceding re-actions. This is remarkably the case in intermittents, which Cullen therefore has selected as illustrative of his theory of fever. This tendency to remission and exacerbation, is principally owing to the loss of balance between the vital principle of the arterial and that of the venous blood. When this is restored, the paroxysms become less frequent and violent, and the febrile symptoms gradually subside.

As nervous influence is essential to the support of vascular action, and as the heart and arteries are deprived of this influence in consequence of visceral congestions, &c. which prevent its development, particularly if the brain is one of the organs so affected, re-action might not be excited, or only to a partial extent; and, therefore, defective re-action characterises the worst kind of fevers: or, if febrile excitement be well-marked, the expenditure of nervous energy becomes so much augmented, in order to support the increased vascular action, that exhaustion must ultimately occur; the approach of which is indicated by the symptoms characterising the third stage, which soon terminates in death, if the causes which prevent a proportional replenishment of nervous energy be not removed.

The phenomena of inflammation admit of explanation on similar principles. Inflammation consists in arterial fulness, and its symptoms, like those of febrile re-action, are always preceded by diminished nervous energy; the development of which is either directly obstructed by the exciting cause acting on the nerves themselves, or indirectly, as when it is the effect of venous congestion. Re-action here is likewise induced, and differs from what occurs in fevers only in being local and continued; although, if the inflammation be great, or affect any important organ, fever becomes established, and continues till the exciting cause be removed. This is what Hunter calls *sympathetic fever*, and is characterised by a general state of excitement, with daily exacerbations; and exhaustion does not occur till, by protracted vascular action, or disorganisation produced by the inflammation, the expenditure of nervous energy becomes greater than its re-production. The energies of the system are much weaker in idiopathic fever, than in that which is the effect of inflammation; and, although inflammation is a frequent concomitant of continued fevers, yet, as it is only an effect of febrile action already established, it does not alter their type.

When inflammation occurs externally, the continued excitement by which it is characterised, is generally owing to some evident mechanical cause impeding the evolution of nervous energy; as in burns, which often wholly destroy the function of the nerves, and mortification ensues, without the intervention of re-action; or in common wounds or contusions, which are accompanied with a considerable reduction of temperature previous to the occurrence of inflammatory excitement; an increased flow of blood to the part follows, evinced by redness and swelling. This is accompanied with a corresponding evolution of nervous energy, and the nerves are thereby rendered morbidly sensible. Increased sensibility seems, therefore, produced by two causes: 1, pressure occasioned by the distended vessels on the contiguous nervous filaments; 2, increased development of nervous energy, produced by arterial fulness. A curious fact observed by M. Magendie, in his late experiments on the fifth pair of nerves, strikingly illustrates the connexion which exists between the loss of nervous sensibility and inflammatory action. "Twenty-four hours after the division of the fifth pair of nerves, the cornea begins to become opaque; at the end of seventy-two hours, it is much more so; the opacity increases; and five or six days after the operation, it is as white as alabaster. From the second day, the conjunctiva becomes red, appears inflamed, and secretes a very abundant milky, puriform matter; the eyelids are either wide open or motionless, or else they are sealed by the puriform matters which have dried between their edges; and, when they are separated, a considerable quantity of matter, as above described, escapes. Towards the second day after the section, the iris is likewise observed to become red, its vessels are developed, and, in fine, the organ inflames."

When inflammation is once excited, from whatever cause produced, it consists solely in increased arterial action, in which the powers of life seem preternaturally augmented and expended. The difference between arterial excitement and venous congestion, is particularly exemplified by phrenitis and typhous fever. In the former, the post-mortem appearances of the brain show the arteries of that organ to be unusually distended with blood. In the latter, the veins are found in a state of congestion, and gorged with black blood. The general state of excitement characterising phrenitis, is evinced by increased vascular action, loud and boisterous delirium, shouting, singing, &c. In typhus, the energies of life seem torpid; vascular action, although morbidly increased, is accompanied with a general loss of power; the delirium is low and muttering, or the patient is affected with stupor. As venous blood contains less of the vital principle than arterial, the supply of nervous energy from the brain is diminished in venous congestion of that organ; its functions are deranged, and the energies of the system are weakened. In arterial fulness, or inflammation, on the contrary, the sensorial functions are deranged from the too-abundant production of nervous energy, which causes the violent symptoms characterising phrenitis. If the inflammation is not subdued, these symptoms continue till, by reiterated action, the expenditure of nervous energy becomes greater than its reproduction, or till the functions of the brain are so far impeded by depositions or disorganisations, as to prevent the further development of nervous energy, and the opposite state of exhaustion or collapse, and ultimately death ensues.

That the blood possesses vitality,—that the vital principle is in greater proportions in arterial than venous blood,—that the energies of the system are derived from the blood, through the medium of the brain and nerves,—are the facts on which the preceding pathological views are founded, and are supported by the phenomena exhibited in *animal existence*, *the mode pursued by nature in supplying the various organs with blood*, &c.

*Animal existence.*—During infancy there appears an exuberance, if we may be allowed the expression, of vitality: that period of life is peculiarly characterised by activity and sprightliness; and, in young animals, we observe a disposition to be almost constantly in motion. To support the growth of the body, the operations of life are in active exercise. The various organs have not only to perform their peculiar functions, but are becoming daily more developed, till they have attained maturity. A greater degree of vitality is, therefore, necessary during infancy than at any other period of life; and accordingly we find nature has provided against this exigency, by establishing arterial plethora, which is characteristic of the infantile constitution; and thus the source from whence the energies of the system are derived, is proportioned to their expenditure. As age advances, arterial plethora gradually decreases till full growth is attained, and is attended by corresponding changes in the individual, as well moral as physical. Maturity is established by an equilibrium, which is supposed to exist between the arterial and venous circulations. This continues till middle life, when venous plethora commences, and, as venous blood possesses less of the vital principle than arterial, the energies of the system become weakened. Venous plethora gradually increasing, the powers of life proportionably recede; the infirmities of old age approach; and ultimately death occurs, as the inevitable consequence of the progress of life.

*The arrangement pursued by nature in supplying the various organs with blood.*

It is remarkable that those parts which are of most importance in the animal economy, are supplied with blood either from the aorta or its immediate branches; which circumstance is an additional evidence that arterial blood is the fountain of life, and that it possesses the vital principle in a degree proportioned to its proximity to the heart. Thus, we find the coronary arteries which support the wonderful energies of the heart, arise immediately from the aorta at its commencement. The brain receives its blood from the internal carotids; and it is remarkable that, although the external carotids send off numerous branches to supply the external parts of the head, the internal do not ramify till they have entered the cranium to supply the brain, whose energies they are destined to support. Haller observes, "In every pulsation a great quantity of blood is sent to the brain, equal to the sixth part, or more, of the whole blood in the human body, and conveyed by trunks which arise very near the heart, and from the convexity of the aorta. It is, therefore, not improbable that the strongest parts of the blood, and those most retentive of motion, go to the head." The ophthalmic arteries, which supply the visual organs, are derived from the internal carotids, after they have perforated the dura mater. The pulmonary artery is derived from the heart, and soon divides into two branches to supply the lungs. The chylopoietic viscera are supplied from the cœliac, the first branch of the abdominal aorta, and the mesenteries, which proceed from the latter as distinct branches. From the aorta, also, the renal arteries arise; and, that the organs of generation might likewise be supplied with the purest blood, the spermatic arteries are derived directly from the same trunk, and are obliged to take a long course before they can reach the organs whose functions they are intended to support.

The liver and spleen are also subservient to this law of the animal economy. The spleen, whose use appears involved in so much mystery, might be considered a reservoir of blood, which is peculiarly endowed with vitality. The splenic artery is a principal branch of the cœliac, and subdivides, in the substance of the spleen, into innumerable ramifications, which terminate in the veins without the intervention of cells,—the usual mode of connexion between arteries and veins. The splenic vein unites with the superior and inferior mesenteries to form the vena porta. In consequence of the splenic blood not having to support the function of secretion, its vitality is not expended; and, from the direct communication between the arteries and veins, there seems little doubt but the venous blood of the spleen is as much endowed with vitality as arterial; and, from the short route the blood takes before it enters the vena portæ, it becomes instrumental in the secretion of bile, or at least renders the secretion more perfect. At the same time, it might greatly assist in enabling the stomach to perform its important functions, by supplying this organ with blood strongly impregnated with vitality, which it does through the medium of the arteriæ breves, large branches of the splenic artery, which are distributed on the great curvatures of the stomach. The anomaly of a vein performing the function of secretion, as is the case with the vena portæ, is thus satisfactorily explained, and the spleen assumes that important character in the system to which, from its size, it appears entitled.

[To be continued.]

